# Using Multiscale Molecular Modeling to Analyze Possible NS2b-NS3 Protease Inhibitors from Philippine Medicinal Plants

**DOI:** 10.3390/cimb46070451

**Published:** 2024-07-18

**Authors:** Allen Mathew Fortuno Cordero, Arthur A. Gonzales

**Affiliations:** Department of Chemical Engineering, University of the Philippines Diliman, Quezon City 1101, Philippines; afcordero1@up.edu.ph

**Keywords:** molecular modeling, computer-aided drug design, dengue virus, NS2b-NS3 protease, phytochemicals

## Abstract

Within the field of Philippine folkloric medicine, the utilization of indigenous plants like *Euphorbia hirta* (*tawa-tawa*), *Carica papaya* (*papaya*), and *Psidium guajava* (*guava*) as potential dengue remedies has gained attention. Yet, limited research exists on their comprehensive effects, particularly their anti-dengue activity. This study screened 2944 phytochemicals from various Philippine plants for anti-dengue activity. Absorption, distribution, metabolism, excretion, and toxicity (ADMET) profiling provided 1265 compounds demonstrating pharmacokinetic profiles suitable for human use. Molecular docking targeting the dengue virus NS2b-NS3 protease’s catalytic triad (Asp 75, Ser 135, and His 51) identified ten ligands with higher docking scores than reference compounds idelalisib and nintedanib. Molecular dynamics simulations confirmed the stability of eight of these ligand–protease complexes. Molecular Mechanics/Poisson–Boltzmann Surface Area (MM/PBSA) analysis highlighted six ligands, including veramiline (−80.682 kJ/mol), cyclobranol (−70.943 kJ/mol), chlorogenin (−63.279 kJ/mol), 25beta-Hydroxyverazine (−61.951 kJ/mol), etiolin (−59.923 kJ/mol), and ecliptalbine (−56.932 kJ/mol) with favorable binding energies, high oral bioavailability, and drug-like properties. This integration of traditional medical knowledge with advanced computational drug discovery methods paves new pathways for the development of treatments for dengue.

## 1. Introduction

Over the past five decades, infections caused by the dengue virus (DENV) have increased thirtyfold globally. Annually, an estimated 50 to 100 million new infections occur in over 100 endemic countries, placing approximately 2.5 billion people at risk of contracting the disease [1]. In the Philippines alone, a significant rise was observed, with 220,705 dengue virus type II (DENV-2) cases reported from 1 January to 17 December 2022, marking an 182% increase from the 78,223 cases in 2021. Given these statistics, controlling and researching DENV has become a global health priority [2].

Despite extensive research at both industrial and academic levels, there are currently no clinical drugs available that act directly against DENV, apart from the CYD-TDV vaccine (marketed as Dengvaxia), which was approved in 2015. [1]. This vaccine is deemed safe and beneficial for public health in regions where approximately 70% of the eligible age group shows seroprevalence. However, it is recommended only for individuals who have not previously been infected with DENV [3]. Additionally, the vaccine has faced controversies due to its potential risks of severe dengue in seronegative individuals. Beyond Dengvaxia, other treatments focus primarily on supportive care, such as hydration and pain relief, rather than antiviral therapy.

The limitations of current treatment options highlight the urgent need for safer and more effective anti-dengue agents. The absence of direct-acting antiviral drugs leaves a significant gap in the management and control of dengue infections, particularly in preventing severe forms of the disease and reducing mortality rates. The development of new therapeutic strategies is crucial to address these limitations and improve patient outcomes.

In silico approaches, which include molecular docking and molecular dynamics simulations, offer significant advantages in drug discovery and design. These computational methods allow for the rapid screening of vast chemical libraries, identification of potential drug candidates, and detailed analysis of their interactions with biological targets at the molecular level. The ability to simulate the dynamic behavior of drug–target interactions provides insights into the stability and efficacy of potential inhibitors, which can streamline the drug development process and reduce the need for extensive in vitro and in vivo testing [4].

Recent studies, such as the one by Uday et al. [5], have identified that drug molecules like idelalisib (IDE) and nintedanib (NIN) can effectively bind to the active site of the NS2b-NS3 protease, a critical enzyme in the DENV replication process. This protease’s active site is formed by the catalytic triad of His51, Asp75, and Ser135, which play essential roles as a nucleophile, a base, and an acid, respectively, during peptide bond cleavage [6]. The interaction of these drug molecules with the protease not only alters the enzyme’s dynamics and conformation but also highlights the potential of the catalytic triad as a target for antiviral drugs.

The NS2b-NS3 protease is integral to the viral replication process, cleaving the viral polyprotein at specific junctions to release individual functional proteins necessary for the virus’s lifecycle. The structural basis for the activation of this protease has been well documented. For instance, Erbel et al. provided crystal structures of the NS2b-NS3 protease complex, emphasizing the role of the NS2B cofactor in stabilizing the protease and enabling its catalytic activity [7]. Additionally, Da Silva-Junior et al. demonstrated the critical interactions between the protease and its inhibitors, showcasing the importance of targeting this enzyme in antiviral strategies [8].

Considering the need for effective antiviral agents and the limitations of current treatments, this study turns to the rich biodiversity of the Philippines, exploring the antiviral potential of phytochemicals from local medicinal plants. Research has indicated that approximately 30 different plant species, including *tawa-tawa (Euphorbia hirta*), *papaya* (*Carica papaya*), guava (*Psidium guajava*), *sambong* (*Blumea balsamifera*), and bitter melon (*Momordica charantia*). The leaves of these plants are used in traditional decoctions to alleviate viral infections and related symptoms [9]. This study further investigates 2944 ligands from the phytochemicals present in these plants to assess their effectiveness against DENV through molecular dynamics simulations, virtual screening, and molecular docking. This approach confirms the active site residues and their interactions and has led to the identification of potential anti-dengue drug candidates.

This study primarily focuses on targeting the NS2b-NS3 protease. Virtual ADMET screening, molecular docking, and molecular dynamics (MD) simulations were employed to identify the protease’s most effective inhibitory binding energies. The interactions between small molecule inhibitors with the highest binding energies were obtained and systematically analyzed. The major and minor interaction points within the binding site were demonstrated through a combination of ligand-based and structure-based approaches. Analysis of the results confirmed the interactions with the active site residues and the discovery of potential anti-dengue drug candidates. This approach offers a new strategy for designing novel and specific therapeutic agents against the dengue virus that are safe for human consumption.

To our knowledge, this study represents the use of the largest virtual phytochemical library from Philippine medicinal plants to date to inhibit the DENV NS2b-NS3 protease. This research aims to explore bioactive compounds and possible mechanisms of action that could pave the way for developing new antiviral drug leads from these plants, providing a novel, significant solution to a pressing global health issue.

## 2. Materials and Methods

The crystallized 3D structure of NS2b-NS3 protease from the DENV-2 serotype was obtained from the Protein Data Bank (PDB) of the Research Collaboratory for Structural Bioinformatics (RCSB), PDB ID: 2FOM. The protein structure was utilized directly from the wild dengue virus type 2 (DENV II) without any attached ligands, as referenced in the study of Yildiz et al. (2013) [10].

Before docking experiments, the protein crystal structure was prepared by adding necessary hydrogen atoms, optimizing hydrogen bonds, and removing solvents and metals/ions using Avogadro [11] and Schrodinger Maestro [12]. The clean 2FOM PDB file was subjected to MODELLER, where a series of Python scripts were used to supply missing residues and refine the generated loops. Since new loops were added, a minimization step was performed using the steepest descent algorithm to ensure that the protein structure was relaxed before the docking experiments [13]. Additionally, missing atoms were addressed using Swiss PDB Viewer to ensure the completeness of the structure [14].

Structures of the reference compounds idelalisib (IDE) and nintedanib (NIN) were downloaded from PubChem (www.pubchem.com) [15]. These drug molecules, primarily used in cancer treatment, were selected as reference compounds based on the findings of Uday et al. (2021) [5]. Ligand structures were obtained from Dr. Duke’s Phytochemical and Ethnobotanical database [16] and the comprehensive phytochemical database of Philippine plant phytochemicals from the UP Diliman Department of Chemical Engineering virtual phytochemical library. This library was also utilized in a previous study by Macalalad and Gonzales (2021), which targeted a different protein and disease [17]. All structures were optimized using the energy minimization tool of Maestro [12] to enhance ligand structures for protein binding, improve the accuracy of docking results, and increase the efficiency of computational screening methods.

### 2.1. Test for Pharmacological Viability

Biocompatibility refers to the ability of a substance or material to interact with living cells, tissues, or organs without causing harm or adverse reactions. Biocompatibility is crucial in drug development because drugs are designed to interact with specific targets in the human body. Any harmful effects on healthy cells or tissues can lead to serious side effects and health complications.

One way to ensure the biocompatibility of drug candidates is to test for their chemical absorption, distribution, metabolism, excretion, and toxicity (ADMET) using computational tools like SwissADME (www.swissadme.ch, accessed on 22 January 2023) [18] and Chembioserver (https://chembioserver.vi-seem.eu, accessed on 22 January 2023) [19]. SwissADME [18] is a web-based platform that allows researchers to predict the pharmacokinetic properties of small molecules, such as drug candidates, based on their chemical structures. Here, Lipinski [20], Ghose [21], Veber [22], Egan [23], and Muegge [24] filters were applied, as they predict the drug-likeness of a compound with a specific biological activity designed for the oral route of administration. Lipinski filter recommends molecular weight (MW) of less than 500, lipophilicity (expressed as the logarithm of the partition coefficient between water and 1-octanol, log P) value of less than 4.15, and the number of O and H atoms not exceeding ten each. The Ghose filter defines drug-likeness constraints: calculated log P is between −0.4 and 5.6, MW is between 160 and 480, molar refractivity (MR) is between 40 and 130, and the total number of atoms is between 20 and 70 [21]. Veber filter includes a less selective constraint such as rotatable bonds (RB) of less than ten and total polar surface area (TPSA) of less than 140 Å while Egan recommends log P of less than 5.88 and TPSA of less than 131.8 Å [22,23]. For Muegge’s rule, the constraints are MW between 200 and 600, log P of −2 to 5, TPSA of less than 150 Å, number of rings less than 7, at least four carbon atoms, number of RB less than 15, hydrogen bond acceptor (HBA) less than 10, and hydrogen bond donor (HBD) less than 5 [24].

To further ensure the viability of the compounds, we utilized the ADME properties estimated by SwissADME [18] for the absorption, distribution, metabolism, and excretion (ADME) analysis. The inclusion criteria for the compounds were based on the following ADME properties:

• Absorption: High gastrointestinal (GI) absorption, as predicted by Lipinski and Egan filters;
• Distribution: Suitable bioavailability and favorable blood–brain barrier (BBB) permeability, indicated by passing the Veber and Ghose filters;
• Metabolism: Predicted to be non-substrate and non-inhibitor for major cytochrome P450 enzymes, ensuring minimal metabolic interactions, guided by Lipinski and Muegge filters;
• Excretion: Favorable excretion profiles with reasonable half-life and low potential for accumulation, supported by Lipinski and Muegge filters.

Compounds that met the above criteria were included for further analysis, while those that did not meet these criteria were excluded. For instance, if a compound passes the Lipinski filter, then it can be assumed to have high GI absorption. Likewise, it is also likely to be well absorbed in the human body. If a compound showed suitable bioavailability and passed the Veber filter, it was deemed to be easily distributed. Compounds predicted to be non-inhibitors of cytochrome P450 enzymes were expected to have minimal metabolic interactions, aligning with the Lipinski and Muegge filters. Favorable excretion profiles indicated by the Muegge filter ensured that the compounds had a low potential for accumulation in the body. This rigorous screening ensured that only the compounds with the most promising pharmacokinetic profiles were selected for subsequent molecular docking and dynamics simulations.

The structures of the ligands were also uploaded onto ChemBioServer [19], which can detect certain toxic functional groups and evaluate their toxicity.

### 2.2. Molecular Docking

Molecular docking was carried out using Autodock 4.2 [25], an automated procedure for predicting the interaction of ligands with biomacromolecular targets. This method is designed to assist researchers in determining biomolecular complexes by balancing two conflicting requirements: achieving robust and accurate results while maintaining reasonable computational demands. The ideal procedure would find the global minimum in the interaction energy between the substrate and the target protein, exploring all available degrees of freedom (DOF) for the system. However, it must also run on a laboratory workstation within an amount of time comparable to other computations that a structural researcher may undertake, such as a crystallographic refinement.

To meet these demands, several docking techniques simplify the docking procedure. AutoDock combines two methods to achieve these goals: rapid grid-based energy evaluation and efficient search for torsional freedom [25]. Recent studies have shown that AutoDock 4.2 performs competitively, ranking second in accuracy among various docking programs, demonstrating its reliability and justifying its use for our study [26].

To validate our docking results, we performed a comparative analysis involving the Root Mean Square Deviation (RMSD) of the ligand, glycerol (GOL), from the crystallized protein structure (PDB ID: 2FOM) and the docking result of glycerol on NS2B-NS3 protease using Autodock 4.2. This approach involved measuring the RMSD of the original glycerol structure from the crystallized protein and comparing it to the RMSD of the same ligand docked using Autodock 4.2.

**Figure 1:**
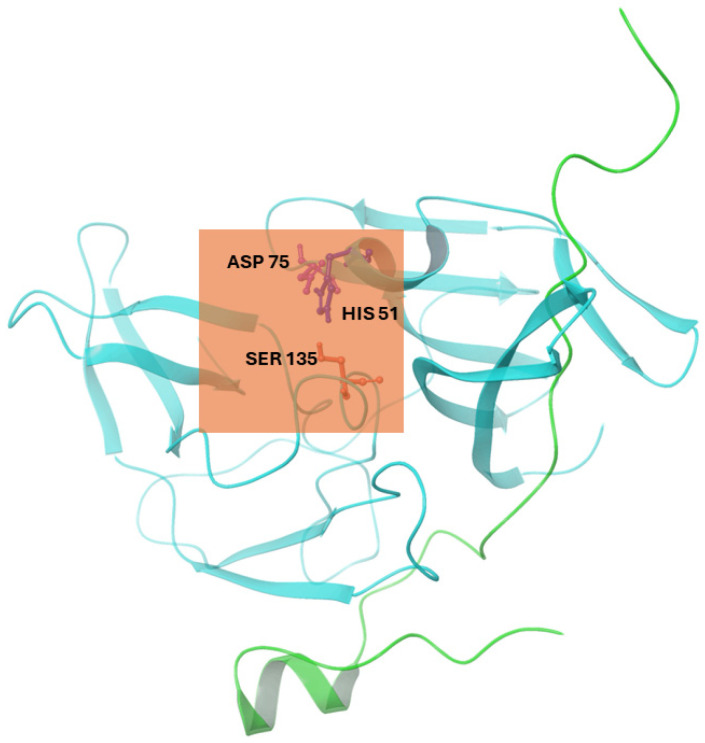
Cartoon representation of the NS2b-NS3 protease (PDB ID: 2FOM) showing the active site grid (orange box) and the catalytic triad residues (ASP 75, HIS 51, and SER 135). Illustration was generated using Schrodinger Maestro [12].

In Autodock 4.2, we implemented a Lamarckian Genetic Algorithm (LGA). Autodock Tools 1.5.6 generated a grid box surrounding the selected residues: His51, Ser135, and Asp75. The grid box also corresponds to the location of the active site, which was determined to be the catalytic triad of the three selected residues [27], [10]. The grid center coordinates for the binding cavity of NS2b-NS3 protease (2FOM) were set to −2.249, −9.192, and 16.146 in the x, y, and z directions, respectively, with a grid box size of 50 × 50 × 50 Å (0.375 Å spacing), as shown in Figure 1. For exhaustiveness, the number of GA runs was set to 1000, with 1000 poses generated per run. After the preparation of grid (.gpf) and docking (.dpf) parameter files, molecular docking was conducted using Cygwin as a Linux-like environment for Windows. From this, only the best conformation (one with the most negative binding energy) per run was selected.

Each complex’s best docking conformations were compared through their binding energies, in which the ten ligands with the highest binding energy were selected. Autodock output (.pdbqt) files were converted to .pdb format through Schrodinger Maestro [12]. The same software was also employed to analyze the interacting residues from NS2b-NS3 protease.

### 2.3. Molecular Dynamics Simulation

Molecular dynamics (MD) simulation evaluates the conformational stability of the complex and predicts any changes in the structure within a specific period. The best conformation of protease and protease–ligand complex systems was subjected to MD simulations with the help of the Groningen Machine for Chemical Simulations (GROMACS) 2021.3 [28]. For the protein, the Chemistry at Harvard Macromolecular Mechanics (CHARMM) 36 force field—July 2022 parameter was used throughout the whole simulation process. On the other hand, the ligand’s force field parameters were generated from the CGenFF (https://cgenff.umaryland.edu/, accessed on 22 January 2023) server of the University of Maryland Baltimore [29].

The CGenFF web server provides accurate force field parameters for small molecules, ensuring compatibility with the CHARMM force field used for the protein. We evaluated the force field parameters obtained from the CGenFF server to ensure they were appropriate for our ligands. This evaluation process involved generating initial force field parameters, including bond lengths, angles, dihedrals, and non-bonded interactions. These parameters were then validated by checking that bond lengths and angles fell within expected ranges for similar chemical groups, verifying that dihedral angles reflected realistic conformational preferences, and confirming that van der Waals and electrostatic interactions were consistent with those observed in similar systems. Additionally, the parameters were tested through a series of energy minimization steps and short initial dynamics simulations (10 ns) to observe any abnormalities or artifacts in the ligand’s behavior.

The ligand–protein complexes were dissolved in TIP3P water, containing 0.15 M NaCl ions (NaCl concentration in human blood), with sodium (SOD) and chloride (CLA) ions used for neutralization. The GROMACS command “genion” was used to add these neutralizing ions, ensuring that the system had a neutral charge before the simulation. A 5000-step structure optimization with the steepest descent algorithm was performed to minimize the energy of the system, followed by a 100 ps equilibration in the NVT and NPT ensembles using V-rescale thermostat and Berendsen barostat for temperature and pressure coupling. Triplicate MD simulations of 100 ns of chemical time were performed with a working temperature of 300 K and a constant pressure of 1 bar (0.9869 atm). Snapshots of the trajectory were saved every 100 ps.

To ensure the accuracy of our MD simulation findings using GROMACS 2021.3, we also performed a validation step. This involved running MD simulations of the reference compounds idelalisib and nintedanib against DENV II NS2b-NS3 protease. We then compared the binding energies of these two compounds to the binding energies reported in the study by Uday et al. [5]. Their reported binding energies were −35.564 kJ/mol for idelalisib and −34.7272 kJ/mol for nintedanib.

### 2.4. Molecular Mechanics/Poisson–Boltzmann Surface Area (MM/PBSA) Analysis

The Molecular Mechanics/Poisson–Boltzmann (MM/PB) method provides a more accurate calculation of the binding strength between ligands than docking experiments. In the Molecular Mechanics/Poisson–Boltzmann (MM/PB) approach, the overall energy of a molecule (Δ*G_binding_*) is calculated as follows:

(1) $$∆G_{binding}=∆G_{complex}-(∆G_{protein}+∆G_{ligand})$$

Here, Δ*G* can be calculated as the sum of its molecular mechanics energy (which considers van der Waals and electrostatic interactions), its polar solvation free energy, and an adjustment for the molecule’s configurational entropy [30]. It can be expressed as follows:

(2) $$∆G=∆E_{vdW}+∆E_{elec}+∆G_{polar}+∆G_{nonpolar}-T∆S$$

Non-polar solvation energy (Δ*G_nonpolar_*) can also be estimated by using the most widely used non-polar model, the solvent accessible surface area (SASA) model. It includes the work performed to create a cavity in the solvent and the attractive forces between the solvent and solute [31].

The MM/PBSA method was implemented through the g_mmpbsa tool [31] of GROMACS with the help of the Python script MmPbSaStat.py. However, the tool cannot calculate the absolute binding energy due to a lack of entropic energy calculations [31].

The MM/PBSA analysis considered only the part of the simulation in equilibrium, specifically the final 10 ns of the MD simulation trajectories. Frames were extracted at regular intervals from this equilibrated portion to ensure an accurate representation of the ligand–protein interactions. A total of 100 frames (10 ns) were used for the MM/PBSA calculation, providing a comprehensive overview of the interaction dynamics. The parameters used for the g_mmpbsa and pbsa.mdp [31] tools included setting the polar calculation to “yes,” adjusting the grid spacing to 0.5 Å, and configuring the solvation models to account for both polar and non-polar contributions accurately. This setup ensured a detailed and precise calculation of the binding free energies for the ligand–protein complexes.

## 3. Results

### 3.1. Biocompatibility and Toxicity Test Results

Phytochemicals or bioactive compounds found in plants are an alternative source of drug candidates, offering a vast array of natural molecules with diverse structures that have yet to be fully explored. Eighty percent of the population in developing countries continues to rely on herbal extracts due to their demonstrated safety and effectiveness in traditional medicine [32].

Table 1 summarizes the results of chemical absorption, distribution, metabolism, excretion, and toxicity (ADMET) tests of 2944 ligands using SwissADME [18] and Chembioserver [19]. To assess the drug-likeness of each ligand, we counted the number of violations on five different filters (Lipinski, Ghose, Veber, Egan, and Muegge) and recorded them in Table S1.

Any compounds that had a total of five or more violations, as recommended by the SwissADME server for an oral drug [33], across all filters were deemed “failed” and were not subjected to toxicity testing. Imposing rigorous screening measures on the compounds is essential to understand their drug-like properties thoroughly. As such, Table 1 indicates that out of the 2944 test ligands, only 1765 have met the criteria set by the server.

The Muegge filter identified the highest number of violations, totaling 3456, with the Ghose filter closely behind at 3251 violations. Both filters incorporate the lipophilicity parameter (log P), which primarily accounts for the ligand violations.

Further screening of the ligands resulted in 1265 ligands passing the toxicity test on Chembioserver.

### 3.2. Docking Results

**Figure 2:**
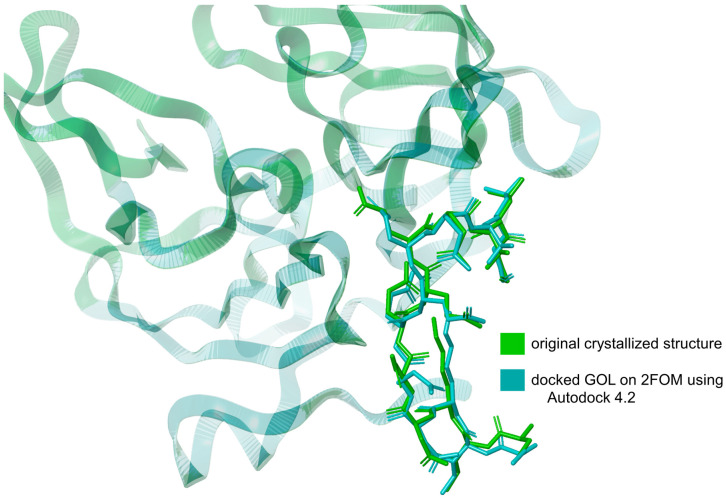
Overlay of the docked glycerol (GOL) structure on 2FOM using Autodock 4.2 and the original crystallized GOL structure from 2FOM. Illustration was generated using Schrodinger Maestro [12].

The validation of the docking method using Autodock 4.2 [25] involved comparing the RMSD of the downloaded glycerol structure from the crystallized protein (PDB ID: 2FOM) to the RMSD of the same ligand docked using Autodock 4.2. The RMSD of the downloaded glycerol structure was measured at 3.432 Å, while the RMSD of the docked glycerol structure was determined to be 3.225 Å. This represents a difference of approximately 6.03%. This relatively small difference in RMSD values indicates that the docking method is valid, as the docked structure closely approximates the original crystallized structure, as shown in Figure 2. The proximity of these RMSD values demonstrates that Autodock 4.2 can reliably reproduce the binding conformation of the ligand within the protein’s active site. Incorporating this validation step ensures the robustness of our docking methodology, providing confidence in the accuracy of the predicted binding interactions between the ligands and the NS2b-NS3 protease.

Table 2 shows the top 10 ligand scores, including the reference compounds idelalisib and nintedanib. All ligands came from plants, with some traditionally used as medicine in other parts of the world, including papaya, bitter melon, guava, basil, neem, cat’s claw, ginger, and lemongrass. The negative scores for all these ligands indicate favorable binding energies in all complexes, which concluded that the protein’s binding site was correctly found and bounded by the ligands. From Table S2, we can observe that among the test ligands, 22 (1.74%) have higher binding energy compared to the reference ligands idelalisib (−25.522 kJ/mol), while 20 (1.58%) exceeded nintedanib (−29.790 kJ/mol).

**Figure 3:**
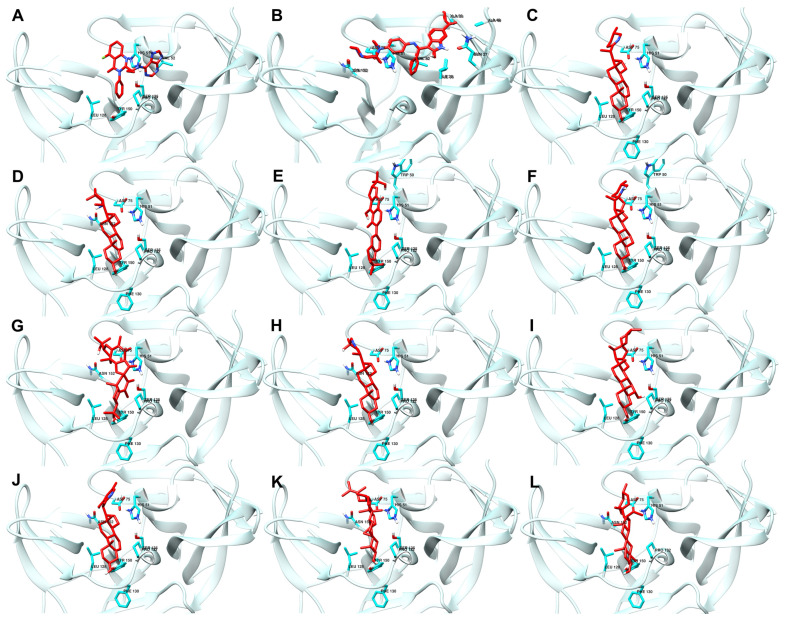
Best docking conformations of top ten ligand–protein complex based on binding energy. Reference ligands: (**A**) IDE; (**B**) NIN. Candidate ligands: (**C**) VER; (**D**) ISO; (**E**) ETI; (**F**) TOM; (**G**) CAR; (**H**) HYD; (**I**) CHL; (**J**) ECL; (**K**) CYC; (**L**) HON. Illustrations were generated using UCSF Chimera 1.16 [34].

Figure 3 shows the best docking conformations of the ligands with the highest binding affinity to NS2b-NS3 protease, including the interacting residues.

### 3.3. Molecular Dynamics Simulation Results

Molecular dynamics (MD) simulations provide detailed insights into the dynamic behavior of molecular systems over time. These simulations are crucial for understanding the stability and conformational changes of protein–ligand complexes. The results from MD simulations can be evaluated using several key metrics, including RMSD, Root Mean Square Fluctuation (RMSF), and Molecular Mechanics Poisson–Boltzmann Surface Area (MM/PBSA) energy analysis. RMSD measures the average deviation of a protein’s atomic positions over time from a reference structure, providing insights into the overall stability of the complex. RMSF assesses the flexibility of individual residues within the protein, highlighting regions of the molecule that exhibit significant motion. MM/PBSA energy analysis estimates the free energy of binding for the protein–ligand complex, offering a quantitative measure of the interaction strength. Together, these metrics provide a comprehensive view of the molecular dynamics and stability of the complexes under study.

To validate the MD simulation method used in this study, we performed simulations using the reference compounds idelalisib and nintedanib against DENV II NS2b-NS3 protease. Our MD simulations yielded binding energies of −37.823 kJ/mol and −39.234 kJ/mol, respectively. The percent difference in binding energies is approximately 6.35% for idelalisib and 13.00% for nintedanib. This relatively small percent difference supports the validity of our MD simulation method. The close alignment of our binding energy values with those reported by Uday et al. [5] indicates that GROMACS 2021.3 can reliably reproduce the binding interactions of known compounds with the NS2b-NS3 protease.

**Figure 4:**
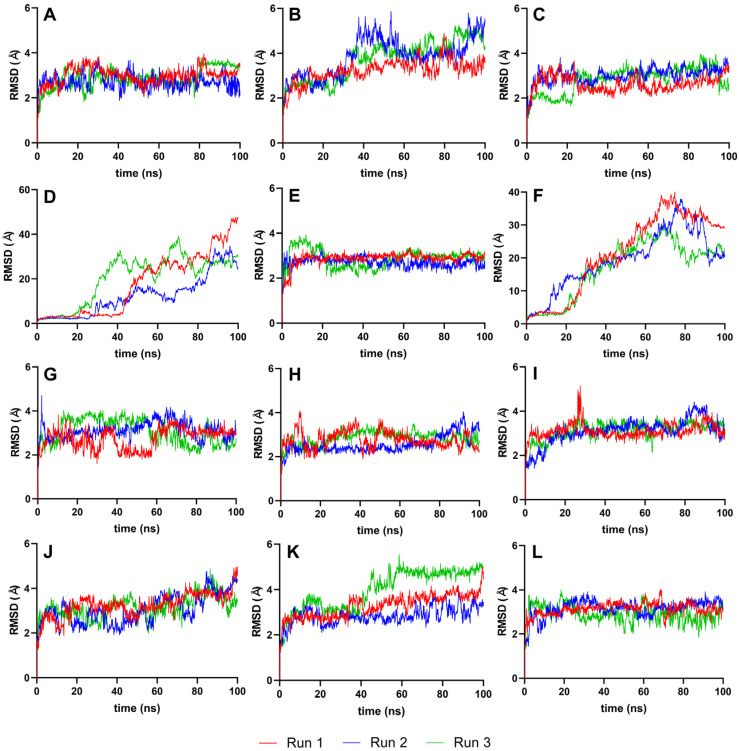
Complex-RMSD of the top ten complexes and two references at 100 ns. Reference ligands: (**A**) IDE; (**B**) NIN. Candidate ligands: (**C**) VER; (**D**) ISO; (**E**) ETI; (**F**) TOM; (**G**) CAR; (**H**) HYD; (**I**) CHL; (**J**) ECL; (**K**) CYC; (**L**) HON. Graphs were plotted using GraphPad Prism 8.2.1 (GraphPad Software, San Diego, CA, USA, www.graphpad.com).

The RMSD analysis used the best ligand–protease complex conformation from molecular docking as the reference structure. Since the docked ligand structures were already minimized and the reference structure exhibited the highest binding energy [35], it was unnecessary to equilibrate the systems further. The 100 ns production run provided adequate trajectories for RMSD calculation, using the protein–ligand complex as the reference and backbone for the least squares fit. Plots from Figure 4 illustrate the RMSD trajectories for the reference and top ten ligands.

Figure 4 details the RMSD plots of the MD simulation for the top ten complexes and reference ligands at 100 ns. Most ligand systems, including VER, ETI, CAR, HYD, CHL, ECL, CYC, and HON, exhibited steady RMSD trajectories with minimal average deviation, indicating high stability. Except for ISO and TOM, all complexes attained equilibrium within the 100 ns simulation timeframe, as evidenced by RMSD values clustering within the 2–3 Å and 4–5 Å ranges.

**Figure 5:**
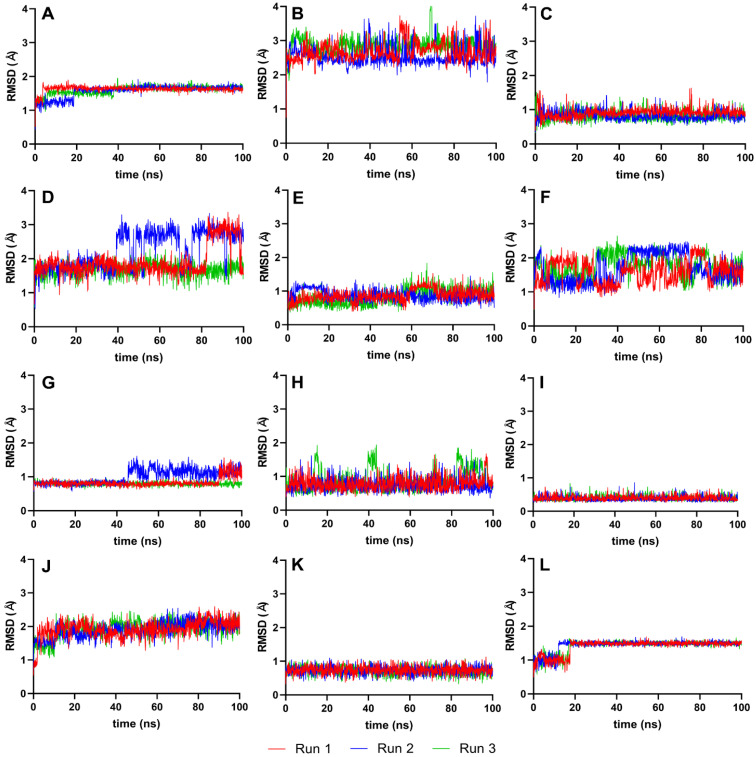
RMSD graphs for the reference and candidate ligands using ligands only as the reference point (Ligand-RMSD). Reference ligands: (**A**) IDE; (**B**) NIN. Candidate ligands: (**C**) VER; (**D**) ISO; (**E**) ETI; (**F**) TOM; (**G**) CAR; (**H**) HYD; (**I**) CHL; (**J**) ECL; (**K**) CYC; (**L**) HON. Graphs were plotted using GraphPad Prism 8.2.1 (GraphPad Software, San Diego, CA, USA, www.graphpad.com).

To further evaluate the conformational stability of the ligands post-docking, we analyzed the RMSD values for the ligands isolated from their complexes. Figure 5 presents these RMSD values, focusing solely on the ligands and using their best-docked pose as the reference. This approach allows us to discern the extent to which each ligand maintains its conformation throughout the simulation. RMSD is a measure of the average distance between the atoms of superimposed proteins at a given time, and in this case, it is applied to ligands. By isolating the ligands, we can better understand their individual stability and how they conformationally behave when not bound to the protease [36].

**Figure 6:**
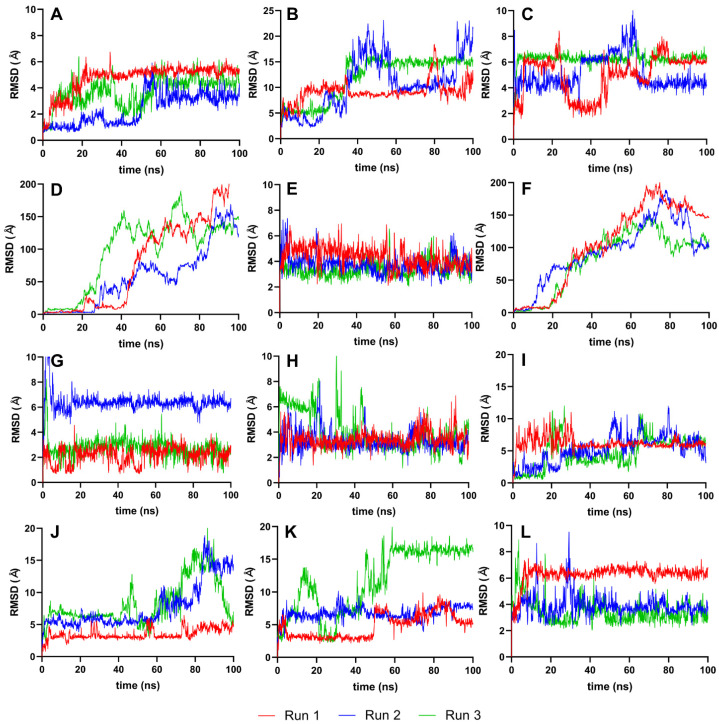
Active site-RMSD of the top ten complexes and two references at 100 ns. Reference ligands: (**A**) IDE; (**B**) NIN. Candidate ligands: (**C**) VER; (**D**) ISO; (**E**) ETI; (**F**) TOM; (**G**) CAR; (**H**) HYD; (**I**) CHL; (**J**) ECL; (**K**) CYC; (**L**) HON. Graphs were plotted using GraphPad Prism 8.2.1 (GraphPad Software, San Diego, CA, USA, www.graphpad.com).

Following the ligand-focused RMSD analysis, we examined the stability of the active site residues of the NS2b-NS3 protease. The RMSD plots in Figure 6 represent the RMSD analysis of the active site residues, Ser135, His51, and Asp75, of the NS2b-NS3 protease complexed with the top ten ligands and two reference ligands over a 100 ns molecular dynamics simulation. This analysis focuses on the key residues involved in the proteolytic activity of the protease, providing insights into how these critical regions maintain their conformation and stability in the presence of different ligands.

**Figure 7:**
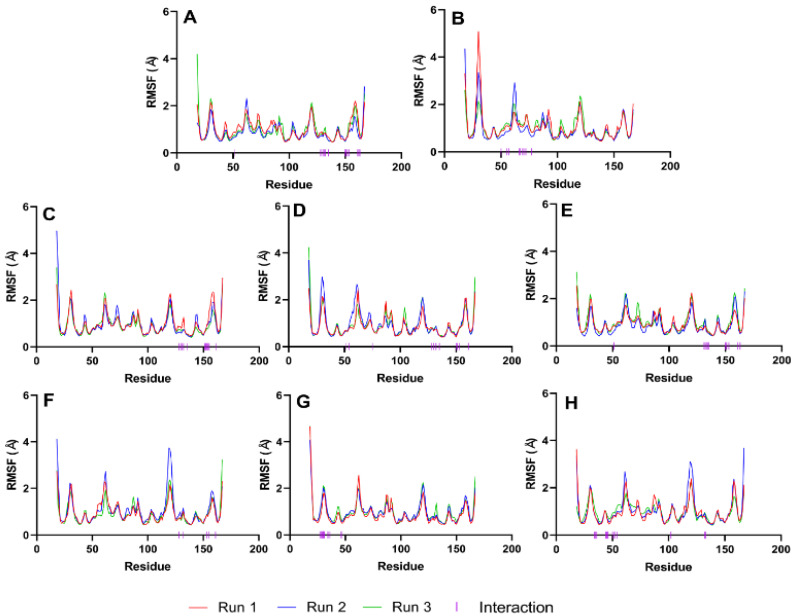
RMSF graphs of NS2b-NS3 Protease docked with the reference and best candidate ligands during the last 10 ns of MD simulation. Residues with interactions were denoted by a purple vertical line (|) on the *x*-axis. Reference ligands: (**A**) IDE; (**B**) NIN. Best candidate ligands: (**C**) VER; (**D**) ETI; (**E**) HYD; (**F**) CHL; (**G**) ECL; (**H**) CYC. Graphs were plotted using GraphPad Prism 8.2.1 (GraphPad Software, San Diego, CA, USA, www.graphpad.com).

Next, we evaluate the flexibility of specific regions within the protein, particularly the active site and gate residues, to gain a more detailed understanding of the dynamics involved. As the structures studied by MD simulations become more complicated, it becomes increasingly common to find high RMSDs related to the large fluctuations of structural subsets that do not reflect the structural changes of the macromolecule. Therefore, calculating the RMSF of the protein is essential to help discriminate between flexible and rigid structures. Figure 7 shows the RMSF plots of the stable ligand–protease complexes (from RMSD analysis) within the last 10 ns of MD simulation. It also shows the interacting residues for each complex.

We proceeded with the MM/PBSA energy analysis to evaluate the binding affinities of the top ten ligands and reference complexes. This method allows us to estimate the binding free energy, providing a quantitative measure of the interaction strength between the ligands and the NS2b-NS3 protease.

The binding affinities of the top ten ligands and reference complexes were determined using MM/PBSA binding free energy analysis. Only the part in equilibrium (final 10 ns) of their MD simulation trajectories was considered here. The results, shown in Table 3, indicate that six ligands (VER, ETI, TOM, HYD, CHL, and CYC) have higher MM/PBSA binding energies than the references, IDE (−38.365 kJ/mol) and NIN (−55.709 kJ/mol). VER still sits on top with −80.682 kJ/mol, followed by CYC with −70.943 kJ/mol. Despite having higher docking scores, CAR and HON have lower MM/PBSA binding energies than NIN. Binding energies for the unstable candidate ligand–protein complex (ISO and TOM) from MD simulations were not determined.

In this analysis, we used the trial with the highest MM/PBSA binding energy as it represents the most energetically favorable binding interaction scenario. This approach highlights the potential of a ligand under ideal conditions, which is often the objective in computational studies to identify the most stable and effective inhibitors. Including statistical measures such as the standard deviation (SD) and standard error of the mean (SEM) helps in understanding the range of binding affinities and the consistency of the binding interactions. The variability observed in the MM/PBSA energies across different runs, as indicated by the SD and SEM values, highlights the sensitivity of the ligand–protein interactions to initial conditions and simulation parameters. This variability is not uncommon in computational studies involving flexible and dynamic systems like protein–ligand complexes. High variance in the results reflects the complex nature of the binding process, emphasizing the importance of considering multiple simulations to obtain a robust assessment of binding energies.

## 4. Discussion

The preceding section provided a summary and observations of the results of each major experimental method. This section delves into the analysis of these results and possible explanations or trends observed.

### 4.1. Biocompatibility and Toxicity Test

Generally, ligands from our database are aromatic hydrocarbons with high molecular weights (MW). Other compounds belonging to various chemical classes, such as flavonoids, quercetins, and natural chalcone (a type of natural ketone) compounds, agree with other studies.

The biocompatibility and toxicity test results reveal several key insights into the properties of the tested ligands. Most phytochemicals have a log *p* value of less than five because they are highly non-polar. Among the parameters measured, it is possibly the least important physicochemical property of a potential drug since most recently discovered and synthesized drug molecules have high log *p* values [37]. When absorbed by the body, the high stability of phytochemicals in gastric and colonic pH also helps absorption and bioavailability [38]. While π-stacking interaction can increase the binding affinity of the inhibitor to its target, de Freitas and Schapira [39] suggest that reducing the number of aromatic rings of a molecule might improve its physicochemical properties, such as solubility.

From Table S1, we can observe that most compounds that failed the toxicity test contain catechol and butanone–Michael acceptor. Catechol is an irritant to the skin, eyes, and respiratory system. Prolonged or repeated exposure to high catechol concentrations can cause skin sensitization and respiratory irritation and may increase cancer risk. On the other hand, limited information is available on the specific toxicity of butanone–Michael acceptor to humans. However, based on the chemical structure of this compound, it is likely to have the potential to cause harm to human health, as it is a reactive compound that can bind covalently to biological molecules such as proteins and DNA, leading to cellular damage and dysfunction. This chemical reaction can disrupt normal cellular processes, potentially leading to adverse health effects [19].

### 4.2. Molecular Docking

Prior to docking, the optimized structures of the references and the top ten ligands can be obtained from Figure S1 of the Supplementary Materials section. These structures reveal important features that contribute to their binding affinities. The reference compounds, IDE and NIN, contain fused aromatic ring systems with nitrogen and oxygen heteroatoms, contributing to their potential for hydrogen bonding and electrostatic interactions. The top ten ligands exhibit a variety of structural components, such as aromatic rings and hydroxyl groups, which facilitate hydrogen bonding, hydrophobic interactions, and electrostatic interactions.

The molecular docking results highlight the binding affinities and interactions of the top ten ligands with the NS2b-NS3 protease. From Table 2, we can observe that the top ten ligands have high molecular weights, resulting in more possible binding sites and interactions with the protein residues. According to a study [1], the molecular surface area of drug-like molecules increases by 95 Å for every 100-unit increase in molecular weight. Thus, higher molecular weight and lipophilicity rather than polar interactions drove most of the potent hits.

The high docking scores observed can also be attributed to the direct attachment of the ligands (RMSD of less than 4 Å) to the protein’s active site, as shown in Figure 3. Specifically, the interactions with the catalytic triad residues (His51, Asp75, and Ser135) are critical for the inhibition of the protease’s activity. These interactions are shown in the figure, demonstrating how the ligands fit into and interact with the active site [1].

Each of the top ten candidate ligands also exhibited at least one hydrogen bond (H-bond) interaction with the NS2b-NS3 protease. Hydrogen bonds play a pivotal role in biological complexes due to their directional nature and significant contribution to molecular specificity [40]. These bonds are crucial in drug design, exploited for their strict geometric and distance constraints, which are essential for achieving high specificity in molecular interactions [39]. Notably, ISO demonstrated the highest number with four H-bonds, while VER, TOM, and CYC each had one. An observation from this study is that NS2b-NS3 residues were more frequently hydrogen bond donors than acceptors.

The combination of hydrogen bonding with the increased number of hydrophobic and polar residue interactions in these ligands could be the key factor contributing to their relatively higher docking scores compared to the reference compounds IDE and NIN. This suggests that the ligands’ ability to establish a diverse range of interactions, particularly H-bonds, enhances their binding affinity, underscoring the importance of considering multiple interaction types in drug design to achieve optimal binding efficiency.

The focus on optimizing binding affinity inherently favored ligands with similar structural features that interact effectively with key residues. The use of a high-throughput virtual screening process and specific docking parameters led to the identification of structurally similar compounds. These compounds share common features that are particularly well suited for binding to the conserved active site of the NS2B-NS3 protease. The NS2B-NS3 protease has a well-defined and conserved active site, which inherently favors certain scaffold structures over others. The catalytic triad and surrounding residues form a specific binding pocket that accommodates ligands with particular structural characteristics. Studies have shown that the conserved nature of this active site limits the diversity of scaffolds that can effectively bind and inhibit its activity [6,7,8,10,41]. Consequently, ligands that fit well within this pocket and interact optimally with the active site residues are likely to share common scaffolds.

The phytochemicals screened in this study are derived from a diverse range of Philippine medicinal plants. Despite the diversity in their sources, the compounds that emerged as top hits may have common pharmacophoric features that are crucial for effective binding. These features include hydrogen bond donors and acceptors, hydrophobic regions, and aromatic rings that align well with the binding site of the protease. Ligand-based drug design approaches often lead to the identification of compounds with similar scaffolds, especially when targeting highly conserved protein structures. In this study, the ADMET screening and molecular docking processes prioritized ligands with favorable pharmacokinetic properties and binding affinities, which have contributed to the convergence of structurally similar compounds.

### 4.3. RMSD Analysis of Protein–Ligand Complex

The stability of most ligand systems, as shown in Figure 4, is indicated by small average deviations (2–4 Å), suggesting a robust interaction between the ligand and the protease, with minimal conformational changes over time. However, ISO and TOM showed a gradual increase in RMSD during the simulation, hinting at ongoing equilibration processes. Significant fluctuations, such as the sharp rise in protein structure deviation at 20–25 ns for ISO and at 60 ns for TOM, were indicative of substantial instability, possibly due to conformational changes within the protein–ligand complex.

Several ligands, including VER, ETI, CAR, HYD, CHL, and HON, achieved stabilization swiftly within the first 5–10 ns of simulation. In contrast, CYC and HON displayed a delayed equilibrium, reaching a stable state at around 60 ns. This behavior may suggest an inherent flexibility and initial adjustment period before the ligands settle into a stable interaction with the protease.

A lower RMSD value relative to the reference structure indicates better reproduction of the correct pose over time [35]. Therefore, smaller ranges and standard deviations in RMSD values are indicative of higher molecular stability. Ligands such as VER, ETI, HYD, CHL, ECL, and CYC demonstrated significantly better stability than the reference ligands, IDE and NIN, as evidenced by their more favorable RMSD values. This improved stability is indicative of a more consistent and reliable interaction between the ligand and the target protein, suggesting a potential for higher efficacy in the intended biological context.

When ligands are docked into the binding site of a protein, it is crucial to assess whether they retain the conformation that is predicted to be optimal for binding. Significant deviations could suggest that the ligand is flexible or that the initial docking pose was not representative of a stable binding conformation. In contrast, low RMSD values, particularly those that remain close to ~0.5 Å, indicate that the ligands maintain a stable conformation, which is often associated with strong and reliable interactions within the binding site [35].

### 4.4. RMSD Analysis of Ligands Only as the Reference Point

Analyzing the RMSD trajectories of the ligands only, as depicted in Figure 5, we observe small movements within the binding pocket for most ligands, suggesting a high degree of conformational stability. This is an essential aspect of drug design, as it reinforces the predictability of the ligand’s behavior when forming a complex with the target protein, thereby increasing the reliability of subsequent analyses and the potential efficacy of the ligand as a drug candidate.

For IDE, ISO, and TOM, the erratic and large fluctuations in their ligand-RMSD could imply that their larger and more complex structures are interacting with elements outside the protein’s binding pocket, leading to less predictable behavior and potentially reducing the binding specificity. These observations might prompt a closer examination of the molecular environment of the binding site or consideration of the ligand’s flexibility during the drug design process to ensure optimal interaction with the target protein.

### 4.5. RMSD Analysis of Active Site Residues and Ligand

The majority of the ligand–protease complexes, particularly those involving IDE (A), ETI (E), CAR (G), HYD (H), CHL (I), and HON (L), exhibited relatively stable RMSD trajectories, clustering within the 2–6 Å range (Figure 6). This minimal deviation suggests robust binding and minimal conformational changes within the active site, which is crucial for effective inhibition. The stable RMSD trajectories for these ligands indicate strong ligand–protein interactions, which aligns with previous research emphasizing the importance of stable binding interactions for effective enzyme inhibition [6].

In contrast, the RMSD plots for VER (C), ISO (D), TOM (F), ECL (J), and CYC (K) showed significant instability within the active site, with sharp rises in RMSD values at various points during the simulation. This instability indicates ongoing equilibration and conformational changes, suggesting weaker and less stable interactions with the active site residues. The erratic RMSD behavior is a strong indicator of poor ligand fit and binding affinity within the active site, which has also been observed in other protease–ligand studies [10,27,42].

### 4.6. RMSF Analysis

RMSF plots provide insights into the flexibility of each amino acid residue during molecular dynamics simulations. These plots are useful for identifying regions of the protein that exhibit significant movement and those that remain relatively stable. As seen in Figure 7, the RMSF values of the residues making up the active site of NS2b-NS3 protease showed minimal alteration. This indicates that the active site residues maintained their structural integrity and did not undergo substantial conformational changes during the simulation period, reflecting a stable interaction between the protease and the ligands. The troughs’ low RMSD values also suggest that the residues had restricted movements, which prevented significant structural changes in the binding site and made it less susceptible to rearrangement. Since the site’s shape remained unchanged during the simulation, ligands could stay inside the cavity and attach themselves completely.

Figure 7 illustrates that loop regions exhibit increased RMSF in comparison to regions forming helices and sheets, suggesting that the presence of secondary structures imparts rigidity to the protein. This rigidity is consequential, as it may influence the protein’s functional conformation and binding potential. Consistent RMSF trajectories across different ligands for the protease (2FOM) indicate a uniformity in interaction patterns with the protein residues, which could reflect a shared binding mode among the ligands.

The analysis highlights critical residues, marked by purple bars on the *x*-axis, which include Asp 75, Ser 135, and His 51 within the active site, as well as Leu 128, Pro 132, and Tyr 161—referred to as gate residues. These residues are characterized by lower RMSF values, demonstrating limited fluctuation and suggesting a stabilization effect in the presence of ligands. Notably, ligands that establish substantial contacts with these active site residues correlate with reduced mobility, implying a direct link between ligand binding and the stabilization of key residues critical for the protease’s activity.

### 4.7. Interacting Residues after MD Simulation

The binding site of the NS2b-NS3 protease exhibited significant interactions with most residues when bound with both the reference compounds and the ten candidate ligands, as detailed in Table 4. This finding is indicative of the higher binding energies observed for the ten complexes compared to the reference compounds [4]. Notably, Leu 128, Pro 132, and Tyr 161 were the most frequently interacting residues, in line with other studies [4,10,27,42,43]. These interactions are critical, as these residues are vital for the enzymatic activity of the protein, suggesting that the ligands may be affecting the protein’s function by mimicking or disrupting its natural substrates or cofactors [4].

**Figure 8:**
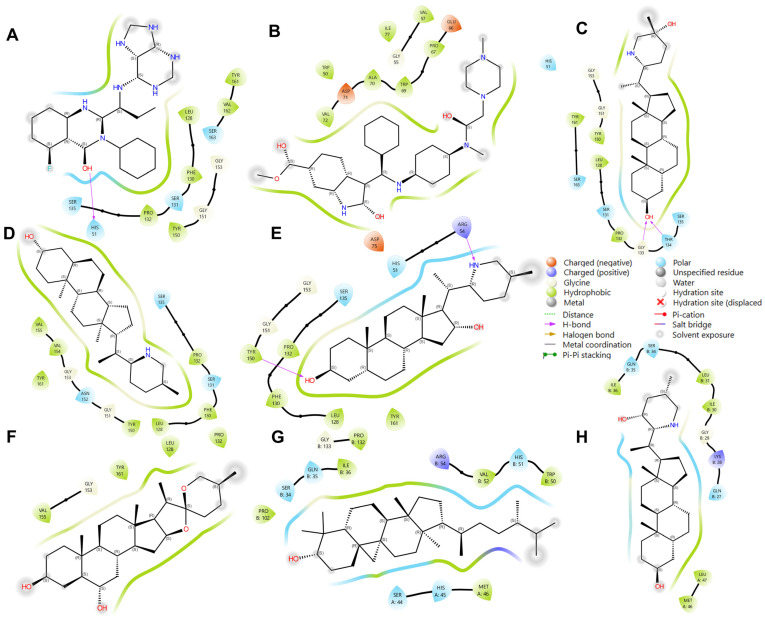
Illustration of interacting residues from 2FOM during the last 10 ns of MD simulation for the top six ligands and references. Reference ligands: (**A**) IDE; (**B**) NIN. Candidate ligands: (**C**) HYD; (**D**) VER; (**E**) ETI; (**F**) CHL; (**G**) CYC; (**H**) ECL. Illustrations were generated using Schrodinger Maestro [12].

In addition to these specific interactions, general types of interactions, such as polar and various electrostatic interactions (Figure 8), also play a significant role in complex stability. The observed interactions were predominantly hydrophobic and polar, with some electrostatic interactions (notably involving Asp 75 and Glu 66). Hydrophobic interactions are known to be crucial in stabilizing protein–ligand complexes, often dictating the overall binding affinity [39]. These interactions, driven by the thermodynamic need to minimize unfavorable water–protein and water–ligand contacts, significantly contribute to the ligand’s specificity and potency.

Most ligands formed hydrophobic contacts with amino acids Leu 128, Phe 130, Pro 132, Tyr 150, and Val 72, with Phe 130 and Pro 132 (Figure 8) being universally present across the top ten ligands. This suggests a key structural motif in ligand recognition and binding. The nature of these interactions, involving aliphatic carbon from the receptor (2FOM) and aromatic carbon from the ligand, highlights the importance of geometric and electronic complementarity in ligand design. The prevalence of aromatic rings in our ligands, consistent with other studies [39,44], underscores the significant role of pi–pi interactions and hydrophobic effects in small molecule recognition. This is further supported by the fact that a large percentage of marketed drugs (76%) contain aromatic rings, with benzene rings being particularly common [44]. These aromatic components likely contribute to the binding efficiency due to their planar structure and ability to engage in pi-stacking interactions with the protein’s active site.

Glycine and phenylalanine emerged as the most common H-bond acceptors. This is likely due to their smaller side chains, which do not obstruct the backbone atoms, thus facilitating the formation of H-bonds. Their increased backbone flexibility also aids in meeting the spatial requirements of H-bonds more effectively [39]. Among the ligands, ISO, ETI, CAR, and CHL formed notable H-bond interactions with critical active site residues such as His 51, Asp 75, and Ser 135. Additional H-bond contacts were observed with other residues, including Leu 128 and Pro 132.

### 4.8. Molecular Mechanics Poisson–Boltzmann Surface Area (MM/PBSA) Analysis

Table 3 shows that CAR (−42.773 kJ/mol) and HON (−54.742 kJ/mol) are only the top ten ligands with lower MM/PBSA scores than NIN. We can also observe that the MM/PBSA energies do not follow the same trend as the binding energy from docking. For example, CYC has a higher MM/PBSA score (−70.943 kJ/mol) compared to CAR (−42.773 kJ/mol), despite having less binding energy (−34.309 kJ/mol to −36.987 kJ/mol, respectively) from docking. This discrepancy can be attributed to the addition of polar and non-polar solvation energies (Equation (2)) in the MM/PBSA method calculation, resulting in more negative binding energy [6].

Molecules with large dipole moments and high polar surface area require more energy in the desolvation process, which would explain why ETI and HYD have high polar solvation energy [30]. In contrast, NIN, VER, and CHL required relatively low solvation energies due to their lower number of polar interactions (Table 3). Given the structural similarity among most ligands (sterols, aromatic), their van der Waals or electrostatic energy was expected to be close. High dipole moments and electrostatic interactions (negatively/positively charged residues) also contribute to the ligand’s electrostatic energy. ETI and HYD have the highest dipole moments among the ten candidate ligands, resulting in increased negative electrostatic energies. Table 3 also shows that HYD has the most positive/negative interactions with the protein, contributing to the higher electrostatic energy.

The electrostatic energy component in MM/PBSA calculations, influenced by factors like dipole moments and hydrogen bonding, plays a crucial role in determining the binding affinity between a ligand and its target protein. High dipole moments and extensive hydrogen bonding significantly contribute to the electrostatic potential energy, enhancing the ligand’s ability to interact with the protein’s active site [30]. Among the ten candidate ligands, ETI and HYD exhibit the highest dipole moments, leading to more substantial negative electrostatic energies. This is indicative of stronger electrostatic interactions with the protein, which is a key factor in stabilizing the ligand–protein complex. Table 3 highlights that HYD forms the greatest number of hydrogen bonds with the protein, further increasing its binding energy. These hydrogen bonds contribute to the overall electrostatic energy and reinforce the specificity and stability of the interaction, providing directional interactions that precisely align the ligand within the protein’s binding site.

For aromatic ligands, the balance between van der Waals (vdW) and electrostatic interactions plays a significant role in the bonding and overall structure of the complexes. Aromatic systems often rely on vdW forces, particularly dispersion interactions, to achieve binding stability. This is due to the electron-rich nature of aromatic rings, which facilitates close packing and effective interaction with the protein’s active site. Despite some ligands, such as VER, CYC, and HON, exhibiting lower electrostatic energies, as noted in Table 3, their substantial vdW energies compensate for this and enhance their overall binding affinities.

The cumulative effect of these interactions, when considered alongside other energy components in the MM/PBSA calculation, reveals that VER and CYC, for instance, achieve binding energies that exceed that of the reference compounds IDE and NIN. This underscores the importance of vdW interactions in stabilizing the ligand within the protein’s binding site, even in scenarios where electrostatic contributions might be less favorable. Additionally, the other seven candidate ligands also demonstrate strong vdW forces, with their energy values closely mirroring those of VER, CYC, and HON. This suggests a general trend where vdW interactions, characterized by their ability to stabilize aromatic ligand–protein complexes without the need for direct electronic interactions, are a critical determinant of binding energy and, by extension, the potential efficacy of aromatic compounds as inhibitors.

**Figure 9:**
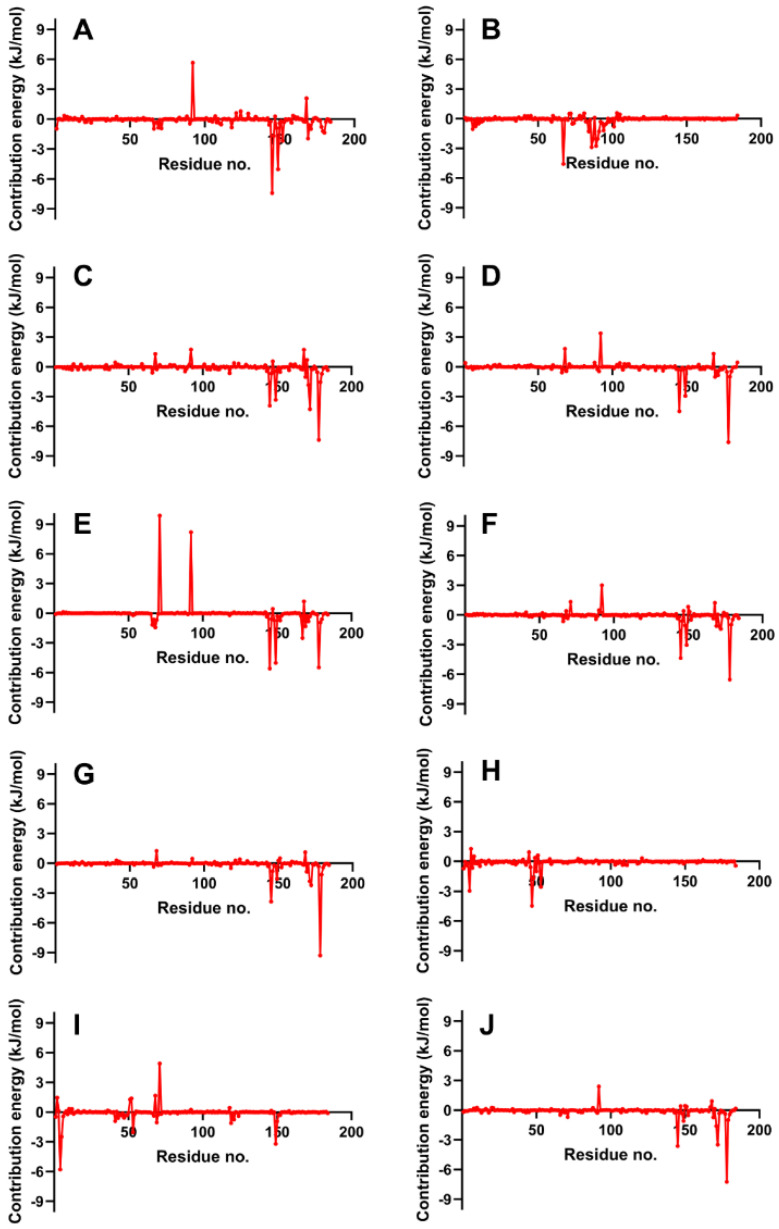
Binding energy decomposition on complexes with the highest MM/PBSA energies. Reference ligands: (**A**) IDE; (**B**) NIN. Candidate ligands: (**C**) VER; (**D**) ETI; (**E**) CAR; (**F**) HYD; (**G**) CHL; (**H**) ECL; (**I**) CYC; (**J**) HON. Graphs were plotted using GraphPad Prism 8.2.1 (GraphPad Software, San Diego, CA, USA, www.graphpad.com).

Figure 9 provides a residue-by-residue breakdown of the binding free energy contributions for the candidate ligands in the NS2b-NS3 protease complex, as determined by MM/PBSA analysis. The analysis highlights the substantial contributions of residues Leu 128, Pro 132, and Tyr 161—designated as gate residues—to the negative binding free energy. These residues are integral to the ligand–protein interaction, acting as crucial elements that govern access to the active site and consequently play a pivotal role in the inhibition of the protease’s replication function. Supporting information from Table S3 offers a detailed view of the individual residue contributions to the binding energies for each candidate ligand, further elucidating their roles in the stability and affinity of the ligand–protease complexes. The active site residues, particularly Asp 75, His 51, and Ser 135, alongside these gate residues, are key in mediating the interactions that lead to potent inhibition, as reflected by the energy contributions captured in the MM/PBSA analysis.

### 4.9. Final NS2b-NS3 Protease–Ligand Complex Structures after Simulation

**Figure 10:**
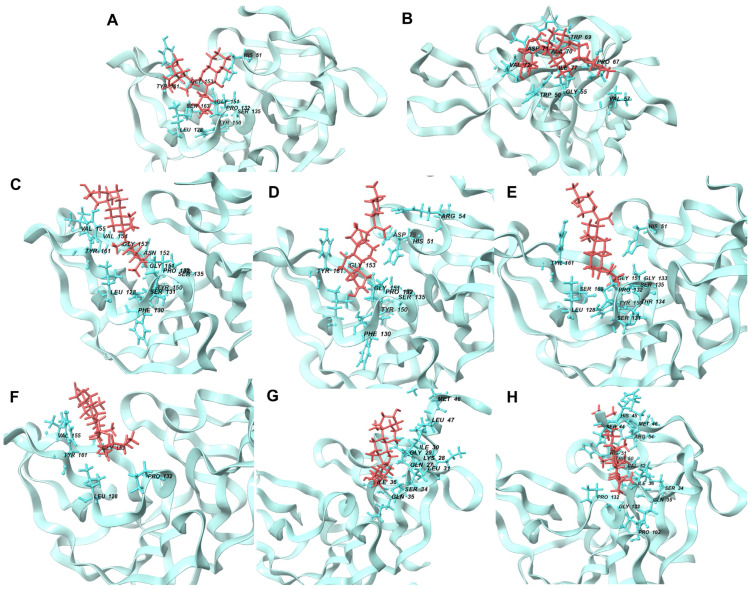
Best conformations of ligand-NS2b-NS3 protein complexes after MD simulation at 100 ns. Reference ligands: (**A**) IDE; (**B**) NIN. Best candidate ligands: (**C**) VER; (**D**) ETI; (**E**) HYD; (**F**) CHL; (**G**) ECL; (**H**) CYC. Illustrations were generated using Schrodinger Maestro [12].

Figure 10 showcases the optimal conformations of ligand–protein complexes post-100 ns molecular dynamics (MD) simulation. The reference ligands IDE (A) and NIN (B) and candidate ligands VER (C), ETI (D), HYD (E), CHL (F), ECL (G), and CYC (H) are illustrated, each interacting within the binding site of the NS2b-NS3 protease. Critical to the functionality of the protease are the catalytic triad residues Asp 75, Ser 135, and His 51, which are central to the enzymatic activity of the protease and serve as key sites for inhibitor binding.

To compare the bound and unbound states of the ligands, we utilized RMSD measurements, as shown in Table 5. The RMSD values indicate the stability of the ligands within the binding site compared to their minimized structures. In molecular dynamics studies, an RMSD value within 2–3 Å is generally considered indicative of minimal conformational changes and high stability in protein–ligand interactions [45]. The stability of these complexes, reflected by RMSD values within this range, demonstrates the reliability of our initial docking predictions.

**Figure 11:**
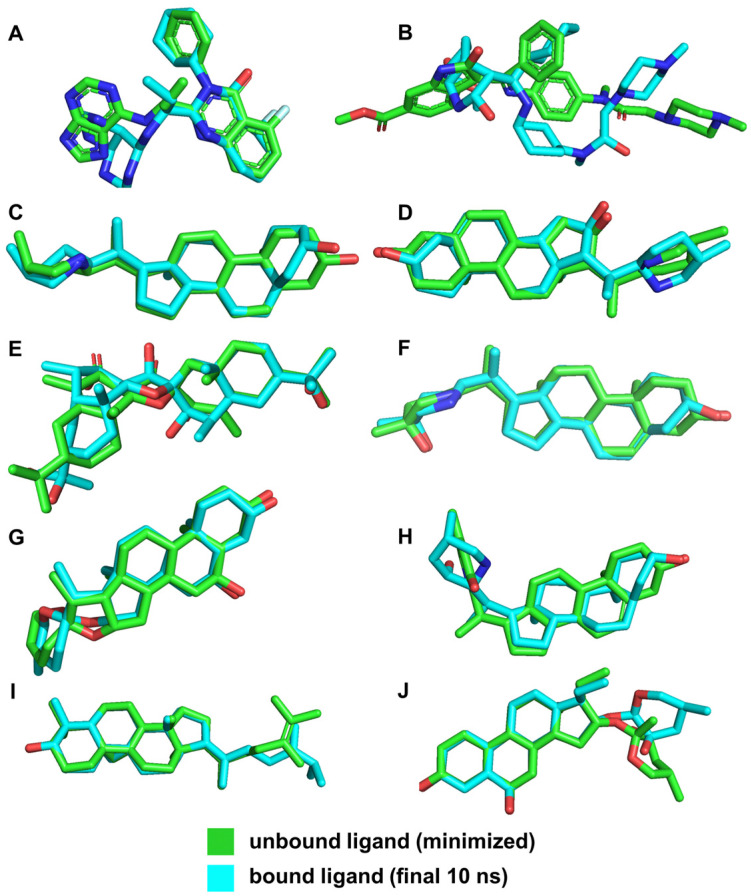
3D structures of ligands for both unbound and bound states. Reference ligands: (**A**) IDE; (**B**) NIN. Candidate ligands: (**C**) VER; (**D**) ETI; (**E**) CAR; (**F**) HYD; (**G**) CHL; (**H**) ECL; (**I**) CYC; (**J**) HON. Illustrations were generated using PyMol 2.5.4 [46].

The low RMSD values for ligands such as VER, ETI, and HON indicate high stability within the binding pocket, suggesting that these ligands maintain stable interactions with the key residues of the NS2b-NS3 protease. This stability is crucial for effective inhibition, as it implies that the ligands are consistently interacting with the active site residues without significant conformational changes. This is further supported by the illustrations in Figure 11.

In contrast, the larger RMSD value for NIN (3.454 Å) suggests significant conformational changes and different interaction dynamics compared to the other ligands (Figure 10). This can be attributed to its distinct binding site and different interacting residues, leading to varied conformational adjustments during the simulation. The different binding interactions observed for NIN, as discussed in the previous sections, involve residues that are not part of the catalytic triad, which account for its larger RMSD and potentially different binding behavior.

By comparing the final 3D structures of the ligand-NS2b-NS3 complexes after the 100 ns MD simulations to their initial docking conformations (Figure 3), several notable observations can be made. First, the overall position of the ligands within the binding site remained consistent, indicating stable binding interactions throughout the simulation period. This stability is crucial as it suggests that the initial docking predictions were robust and that the ligands maintained their interactions with key residues of the protease.

Specifically, the candidate ligands continued to interact closely with the catalytic triad residues Asp 75, Ser 135, and His 51, as seen in the initial docking conformations. This consistent interaction is indicative of the ligands’ potential to inhibit the protease activity by effectively mimicking substrate binding. The hydrogen bonds and hydrophobic interactions observed in the initial docking positions were retained or slightly adjusted, which further reinforces the stability and specificity of these interactions.

For instance, VER and ETI, which exhibited strong hydrogen bonding and hydrophobic interactions in their docking poses, continued to show these interactions post-MD simulation. This was visually confirmed by the consistent position of the ligands relative to the catalytic triad and other key residues, such as Tyr 150 and Pro 132. Additionally, the conformational changes in the protease were minimal, indicating that the presence of these ligands did not significantly alter the overall structure of the enzyme, which is desirable for maintaining the functional integrity of the target protein.

The MD simulation results provided valuable insights into the dynamic behavior of the ligand–protein complexes, affirming that the candidate ligands are well suited as potential inhibitors. The stability of these complexes, reinforced by their energetic profiles, underscores their potential as lead compounds for the development of dengue virus inhibitors, offering promising avenues for future drug development efforts.

## 5. Conclusions

This study proposed that phytochemicals from Philippine medicinal plants could serve as potential inhibitors against the NS2b-NS3 protease of the dengue virus. Our findings identified six promising ligands from five plants, which demonstrated high binding affinities and stability in simulations against the protease.

We assessed the potential of these phytochemicals by evaluating 2944 ligands from phytochemicals found naturally in the Philippines, the largest number of such compounds investigated to date. ADMET analysis showed that 1265 of the compounds are pharmacologically viable for dengue treatment and safe for human consumption. Recent studies also showed that targeting the active site of NS2b-NS3 protease of DENV can inhibit viral replication and prevent viral infection. Idelalisib (IDE) and nintedanib (NIN), which are known drug molecules that can effectively bind to the active site of NS2b-NS3 protease, have been used as references in the study.

Molecular docking experiments were performed in the active site of the protease to find the top ten inhibitors from the 1265 compounds. Results showed that all ten ligands (VER, ISO, ETI, TOM, CAR, HYD, CHL, and HON) have higher docking scores compared to the reference compounds IDE (−25.522 kJ/mol) and NIN (−29.790 kJ/mol). Several interactions between the ligands and active site residues contributed to the high docking scores, such as polar and hydrogen bonds near the active site residues Ser 135, Asp 75, and His 51.

The stability of the top ten complexes and references was evaluated by performing 100 ns MD simulations in triplicate. Analysis showed ISO and TOM were unstable based on their RMSD and RMSF trajectories. On the other hand, the references and the other eight candidates have stable complex-RMSD and ligand-RMSD trajectories within the binding region, having an average RMSD of 3.2 Å on the last ten ns of MD simulation. MM/PBSA analysis further verified the binding affinities of the eight stable ligands against NS2b-NS3 protease, with NIN being the reference. Results showed the final six ligands with higher binding energies compared to NIN (−55.709 kJ/mol): VER (−80.682), ETI (−59.923), HYD (−61.951), CHL (−63.279), ECL (−56.932), and CYC (−70.943). CAR from Carissa carandas (carandas plum) and HON from Agave americana (century plant) can be potential dengue NS2b-NS3 protease inhibitors but had a lower MM/PBSA score than the reference drug.

The study identified six phytochemicals with high binding affinities against the NS2b-NS3 protease of the dengue virus: VER from *Veratrum mengtzeanum* (pimacao), ETI from *Lilion martagon* (Turk’s cap lily), HYD from *Eclipta prostrata* (false daisy), ECL from *Eclipta alba*, CHL from *Yucca gloriosa* (palm lily), and CYC from *Euphorbia hirta* (*tawa-tawa*). Results showed that the study might help explain the anecdotal efficacy of such folkloric medicinal plants in the Philippines. Further studies on contemporary pharmacological approaches, including the isolation of active compounds and elucidating the mode of action of antiviral activities, are warranted to validate these findings.

## Figures and Tables

**Table 1:** Summary of biocompatibility and toxicity results for the test ligands.

Source	No. of Ligands	No. of Ligands that Passed SwissADME	No. of Ligands that Passed ChemBioserver 2.0
Comprehensive Phytochemical Database	2944	1765	1265

**Table 2:** Binding energy and MM/PBSA (Molecular Mechanics/Poisson–Boltzmann Surface Area) scores after molecular docking.

Database No.	3-Letter Symbol	Ligand	Plant Source	MW (g/mol)	Docking Score (kJ/mol)
Reference	IDE	idelalisib	-	415.4	−25.522
Reference	NIN	nintedanib	-	539.6	−29.790
15559023	VER	veramiline	pimacao	509.6	−38.953
10526985	ISO	isolupinisoflavone	banyan fig	438.5	−38.786
168872	ETI	etiolin	Turk’s cap lily	413.6	−38.242
65576	TOM	tomatidine	tomato	415.7	−37.865
101316738	CAR	carindone	Carandas plum	512.7	−36.987
10549683	HYD	25beta-Hydroxyverazine	false daisy	413.6	−35.815
12303065	CHL	chlorogenin	palm lily	432.6	−34.936
10692897	ECL	ecliptalbine	false daisy	409.6	−34.727
39	CYC	cyclobranol	*tawa-tawa*	440.7	−34.309
52931465	HON	hongguanggenin	century plant	464.7	−33.012

**Table 3:** MM/PBSA binding energies of stable candidate ligands and reference.

Ligand	Van der Waals Energy (kJ/mol)	Electrostatic Energy (kJ/mol)	Polar Solvation Energy (kJ/mol)	SASA (kJ/mol)	Binding Energy * (kJ/mol)	Mean Binding Energy †	SD †	SEM †
IDE	−92.068	−17.2	85.545	−14.642	−38.365	−33.82	4.04	2.33
NIN	−78.447	−1.298	34.100	−10.063	−55.709	−43.21	12.53	7.23
VER	−104.94	−6.494	43.497	−13.195	−80.682	−53.67	24.21	13.98
ETI	−107.656	−32.462	94.027	−13.832	−59.923	−52.93	10.26	5.93
CAR	−80.502	−2.056	50.939	−11.154	−42.773	−41.35	1.30	0.75
HYD	−103.191	−27.037	81.377	−13.100	−61.951	−54.00	12.50	7.22
CHL	−82.811	−1.823	31.861	−10.506	−63.279	−52.56	13.01	7.51
ECL	−76.444	−9.489	39.667	−10.666	−56.932	−44.71	12.39	7.15
CYC	−111.620	−4.896	60.568	−14.995	−70.943	−61.41	12.46	7.19
HON	−81.870	−9.805	47.377	−10.452	−54.742	−49.97	4.77	2.75

* The binding energy is estimated from the sum of the van der Waal, electrostatic, polar solvation, and solvent accessible surface area (SASA) energies during the last 10 ns of the simulation. Energy values selected are from the run with the highest binding energy. † Mean binding energy, standard deviation (SD), and standard error of the mean (SEM) were obtained from the three different MM/PBSA runs.

**Table 4:** Summary of interactions between protein residues, references, and stable ligands.

Ligand	IDE	NIN	VER	ETI	HYD	CHL	ECL	CYC
Residue								
Ala 70		**•**						
Arg 54				**•** **•**				**•**
Asn 152			**•**		**•**			
Asp 71		**•**						
Asp 75 *				**•**				
Glu 66		**•**						
Gln 27							**•**	
Gln 35							**•**	**•**
Gly 29							**•**	
Gly 55		**•**						
Gly 133					**•** **•**	**•**		**•**
Gly 151	**•**		**•**	**•**	**•**			
Gly 153	**•**		**•**	**•**	**•**			
His 45								**•**
His 51 *	**•** **•**			**•**				**•**
Ile 30							**•**	
Ile 36							**•**	**•**
Ile 77		**•**						
Leu 31							**•**	
Leu 128 ^							**•**	
Lys 28	**•**		**•**	**•**	**•**	**•**		
Met 46							**•**	
Phe 130							**•**	**•**
Pro 67	**•**	**•**	**•**	**•**				
Pro 102								
Pro 132 ^								**•**
Ser 34	**•**		**•**	**•**	**•**	**•**		**•**
Ser 44							**•**	**•**
Ser 131								**•**
Ser 135*	**•**		**•**		**•**			
Ser 163	**•**		**•**	**•**	**•**			
Thr 134	**•**				**•**			
Trp 50		**•**			**•** **•**			
Trp 69		**•**						**•**
Tyr 150								
Tyr 161 ^	**•**		**•**	**•**	**•**			
Val 52	**•**		**•**	**•**	**•**	**•**		
Val 57		**•**						**•**
Val 72		**•**						
Val 154								
Val 155			**•**					
Val 162			**•**			**•**		

*—denotes residues within the active site; ^—gate residues; •—electrostatic interactions; •—hydrophobic interactions; •—polar interactions; •—hydrogen bond.

**Table 5:** RMSD of the compound in unbound vs. bound states, generated using PyMol 2.5.4 [46].

Ligand	RMSD (Å)
IDE	1.419
NIN	3.454
VER	0.545
ETI	0.598
CAR	1.059
HYD	0.889
CHL	0.177
ECL	1.108
CYC	0.804
HON	0.210

## Data Availability

The data presented in this study are available in the article and the Supplementary Materials.

## Supplementary Materials

The following supporting information can be downloaded at https://www.mdpi.com/article/10.3390/cimb46070451/s1, Table S1: Full ADMET and toxicity results of all 2944 ligands from test for pharmacological viability section; Table S2: Docking results of test ligands and references on NS2b-NS3 protease (2FOM) using Autodock 4.2; Table S3: Decomposition of binding free energy (kJ/mol) on a per residue basis of the complex with strongest MM/PBSA energies. Figure S1: Optimized 3D structures of the references and the top ten ligands. Illustrations are generated using Schrodinger Maestro.
