# Supplementary figures and images for: Using Multiscale Molecular Modeling to Analyze Possible NS2b-NS3 Protease Inhibitors from Philippine Medicinal Plants
Source: Curr Issues Mol Biol. 2024 Jul 18;46(7):7592–618. doi: 10.3390/cimb46070451 (PMC11275823; doi:10.3390/cimb46070451)

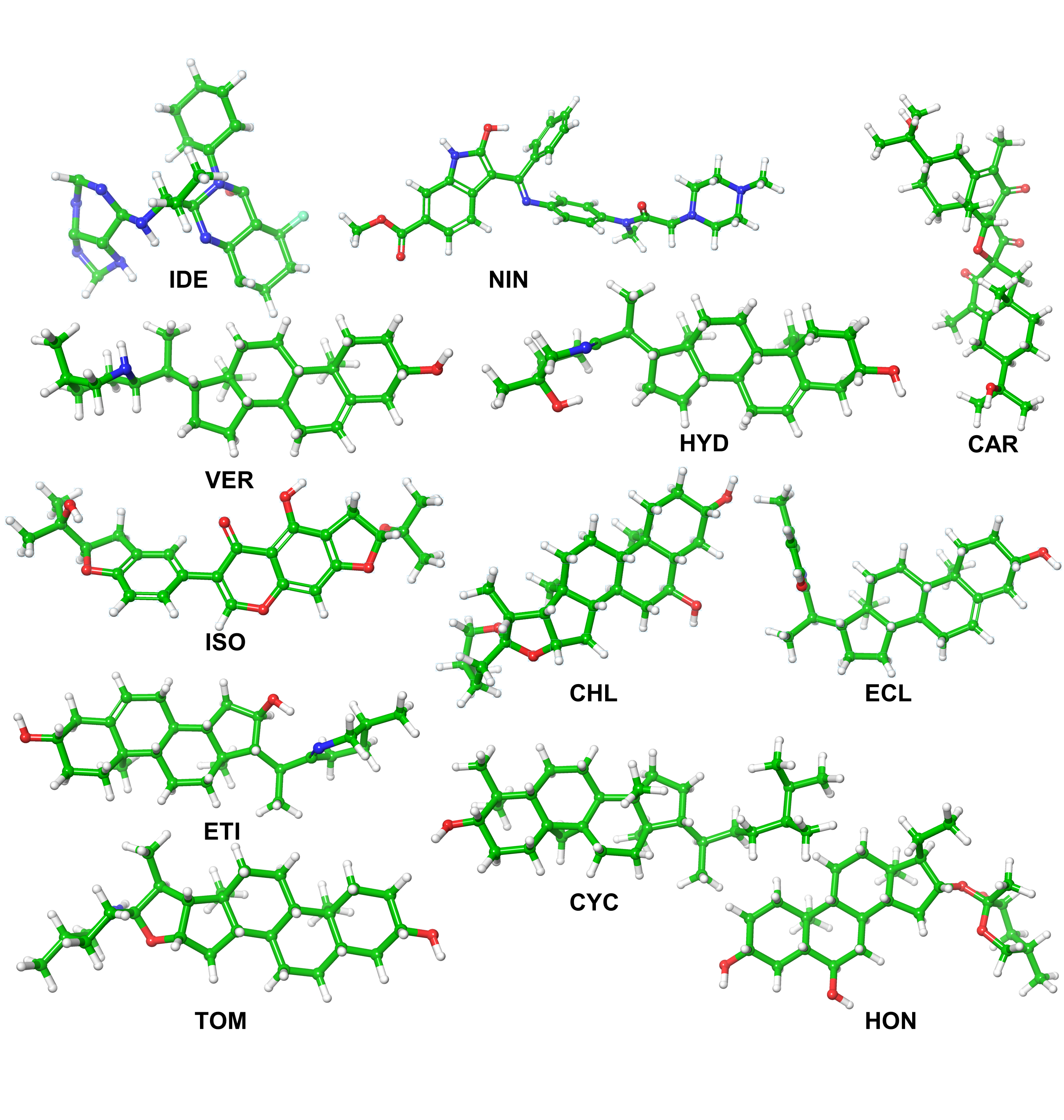

Supplement: Supplementary file 1 [file cimb-46-00451-s001.zip › Figure S1.tif]
